# Study on the microstructure and properties of a laser cladding Fe–Ni–Al coating based on the invar effect

**DOI:** 10.1038/s41598-024-62306-6

**Published:** 2024-05-22

**Authors:** Zhen Wang, Jian Zhang, Fengqin Zhang, Changbao Qi

**Affiliations:** 1 Urban Rail Research Institute, Shandong Polytechnic, Jinan, 250104 China; 2 Key Laboratory of Conveyance and Equipment Ministry of Education, East China Jiaotong University, Nanchang, 330013 China; 3 College of Locomotive and Rolling Stock Engineering, Dalian Jiaotong University, Dalian, 116000 China

**Keywords:** Laser cladding, Invar effect, Al content, Tribology properties, Mechanical properties, Metals and alloys

## Abstract

The purpose of this study was to investigate the effect of Al content on Fe–Ni–Al coatings. A Fe–Ni–Al coating was prepared using a semiconductor laser, and the influence of the Al content on the microstructure and properties of the coating was examined. The microstructure of the coating was characterized using scanning electron microscopy, X-ray diffraction, and energy-dispersive X-ray spectroscopy. The coefficient of thermal expansion of the coating was measured using a static thermomechanical analyzer. The microhardness and wear performance of the coating were analyzed using a microhardness tester and a wear testing machine. The results were as follows. The addition of Al to the Fe–Ni ferroalloy powder resulted in the in situ formation of an AlNi/Fe–Ni laser cladding layer. When the Al content was low, the coating mainly consisted of γ-[Fe,Ni] austenite. As the Al content increased, the matrix phase structure of the cladding layer transformed into the α phase. Consequently, the Invar effect was gradually compromised, leading to the generation of defects in the coating. When the Al content was 4%, the coating performance improved while maintaining a low coefficient of thermal expansion. At this point, there were relatively few cracks in the cladding layer, and it exhibited the best wear resistance.

## Introduction

Laser cladding is an emerging surface strengthening technology that utilizes high-energy density laser beams to melt the surface of a substrate with specific properties, significantly enhancing the surface hardness and wear resistance of the substrate^1,2^. Due to the significant difference in the coefficient of thermal expansion between the cladding material and the substrate, residual stresses are easily generated in the cladding layer during the cladding process, leading to cracking, which significantly reduces the comprehensive performance of the cladding layer. Therefore, reducing the number of crack defects in a cladding layer has become a key objective that urgently needs to be addressed in the field of laser cladding^3,4^.

The Invar effect is a characteristic of Invar alloys. Invar alloys, due to their special Fe‒Ni ratio, exhibit low coefficients of thermal expansion, typically below 1.5 × 10^‒6^ ℃^5–7^. Using Invar alloy powder, a low thermal expansion alloy, as the coating material for laser cladding can significantly reduce the number of crack defects in the cladding layer. However, Invar alloy cladding coating exhibits relatively low strength, hardness, and wear resistance, making it unsuitable for laser surface hardening. Therefore, enhancing Invar alloy coatings is essential^8,9^.

Currently, the methods for strengthening Invar alloys mainly include solid solution strengthening, deformation strengthening, grain refinement strengthening, and precipitation strengthening, but they are mostly applied in casting and metallurgical processes, with limited research reported on their application in laser cladding^10,11^. For instance, Sakaguchi et al.^12^ investigated the precipitation strengthening of super-Invar alloys by intermetallic compounds and revealed that such alloys containing Ti and Si could achieve tensile strengths exceeding 800 MPa during low-temperature annealing. Vinogradov et al.^13^ prepared fine-grained Invar alloys via severe plastic deformation, achieving a significant improvement in the mechanical properties. Compared to those of conventional Invar alloys, the yield stress and fatigue limit increased by threefold and twofold, respectively. These methods can significantly enhance the tensile strength of Invar alloys; however, the processes involved are relatively complex and suitable for the overall heat treatment of castings.

Aluminum, as a highly reactive metal, possesses the ability to react with various elements^14,15^. Zhang et al.^16^ synthesized in-situ Al_3_Ti/AlNi/AlNi_3_/MgNi_2_ reinforced composite coatings on AZ91D magnesium alloy and found that the formation of intermetallic compounds increased the hardness of the coatings. Therefore, the authors attempted to introduce Al into the Invar cladding powder to facilitate chemical reactions between Al and the elements in the Invar alloy, aiming to produce compounds that would strengthen the coating^17^. However, the addition of Al may also potentially disrupt the Invar effect in the coating, affecting the cracking rate of the cladding layer.

In this study, Al was added to Invar alloy powder at concentrations of 2 wt%, 4 wt%, 6 wt%, and 8 wt%. Then, laser cladding experiments were performed. The influences of Al on the microstructure, hardness, and wear resistance of laser-clad Invar alloy coatings were analyzed.

## Materials and methods for the experiment

### Experimental materials

The experimental substrate material is quenched and tempered 45 steel with dimensions of 120 × 120 × 10 mm. Quenched and tempered 45 steel is chosen as the laser cladding substrate for several reasons: first, it has a low cost; second, it is widely available; and third, it exhibits good overall performance. The composition of this steel is mainly iron, and the very low impurity content greatly reduces the number of experimental errors. The substrate undergoes surface roughening and cleaning treatment through a sandblasting process. Afterward, the substrate is thoroughly cleaned with anhydrous ethanol and kept ready for use. No preheating treatment is applied to the substrate. The laser cladding experiment utilizes Fe, Ni, Al, and C powders provided by Nangong Xindun Alloy Welding Material Spraying Co., Ltd., with each element added in different concentrations for the various experimental groups. The compositions of the cladding materials are listed in Table 1. According to the previous research of the authors, increasing the content of 2wt% Al each time in the experiment can better reflect the changes in the microstructure and properties of Fe–Ni–Al.When the Al content exceeds a certain amount, the change is no longer significant.

**Table 1:** Mass fraction of the Fe–Ni-Al cladding powder.

Sample (wt%)	Al	C	Ni	Fe
1 (Invar alloy)	0	0.2	36	Bal
2	2	0.2	36	Bal
3	4	0.2	36	Bal
4	6	0.2	36	Bal
5	8	0.2	36	Bal

**Figure 1:**
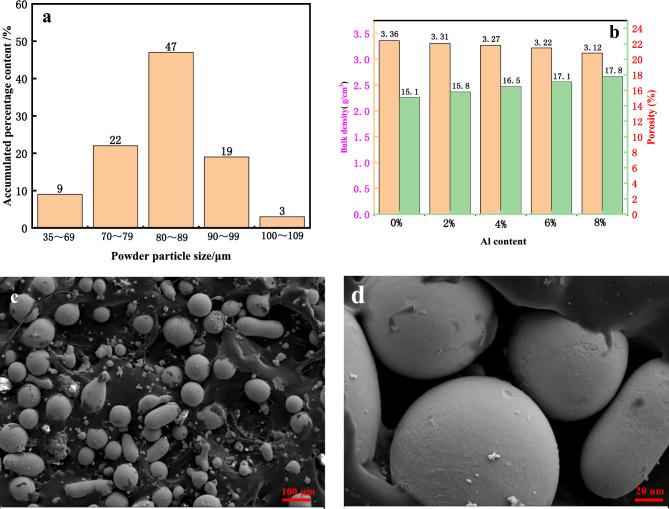
Laser cladding powder completed by ball milling : (**a**) Powder particle size distribution, (**b**) Surface density and internal porosity of powder, and (**c**) Powder morphology.

The prepared powder is milled in an XQM-2L vertical planetary ball mill for 10 h with a ball-to-powder ratio of 2:1 and a milling speed of 250 r/min. The particle size distribution of the milled cladding powder is tested using a laser particle size analyzer, and the results are shown in Fig. 1a. The porosity tester(BSA-DS3) measures the surface density and internal porosity of the clad powder, as shown in Fig. 1b. The morphology of laser cladding powder is shown in Fig. 1c,d. The flowability of the cladding powder is tested using a powder feeder. The feeding accuracy is 99.6%, indicating the excellent flowability of the cladding powder.

### Fe‒Ni‒Al laser cladding coating process

**Figure 2:**
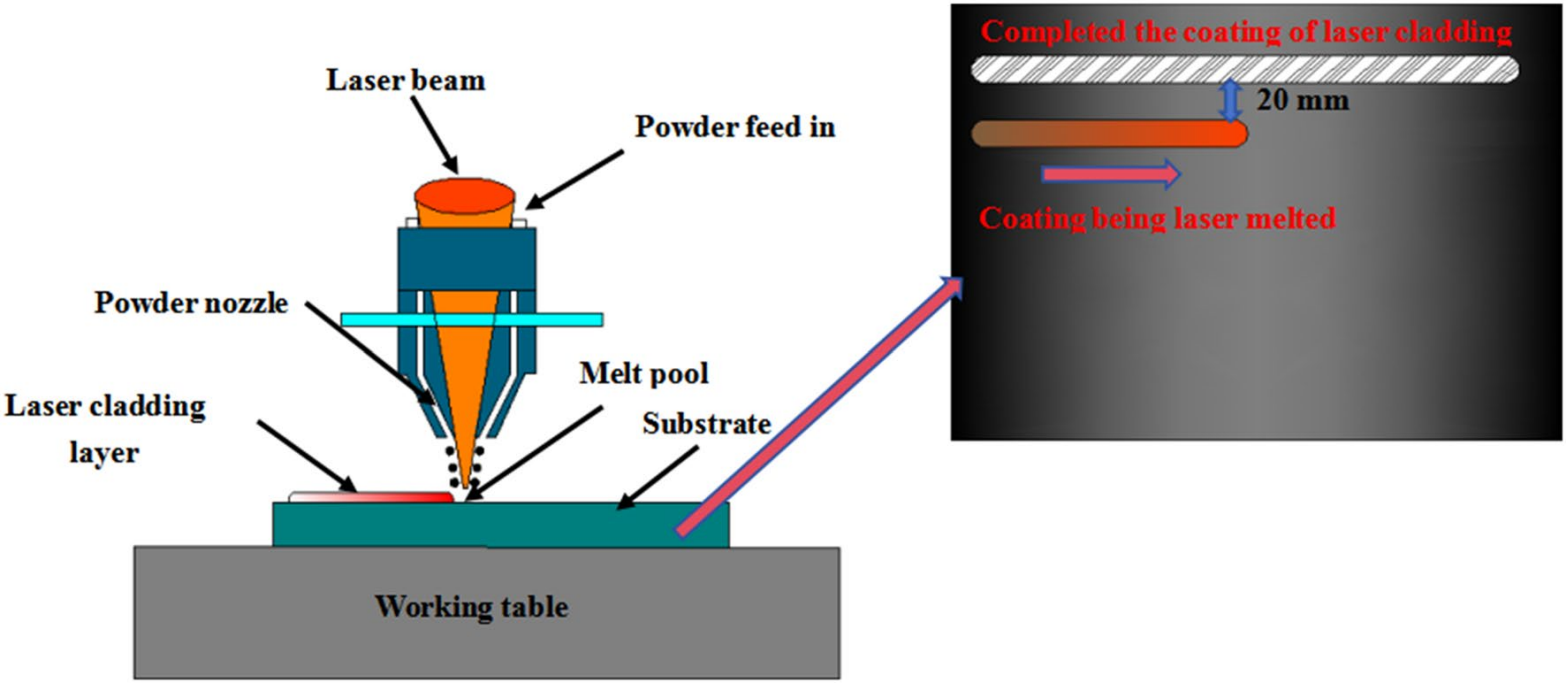
Schematic diagram of the laser cladding working principle.

The experimental setup includes an LDM2500-60 semiconductor laser, an LH-ZSR-1020-02 cladding welding head, and a Herius CC-0715-01 coaxial annular nozzle. Powder delivery is facilitated by a BTSF-2 coaxial powder feeder, with argon gas providing coaxial protection at a flow rate of 15 L/min. The laser cladding process is controlled by a Fanuc M20iD/25 robot. Single-pass, single-layer coaxial powder laser cladding experiments are conducted on the surface of 45 steel, with each experimental group undergoing 5 test repetitions to mitigate experimental variability. The spacing between the cladding layers is 20 mm. The experimental process parameters are detailed in Table 2. The laser cladding process and path is illustrated in Fig. 2.

**Table 2:** Laser cladding parameters.

Laser power (W)	Scanning speed (mm/min)	Powder feed rate (g/min)	Spot diameter (mm)
1400	270	25	3.2

### Microstructural characterization and performance testing

The specimens are cut into cylindrical samples with dimensions of φ4 mm × 20 mm. Afterward, surface grinding and polishing are performed. Subsequently, the samples are cleaned using acetone solution in an ultrasonic cleaner to remove surface oil and impurities. Next, the samples placed in a TMA402F3 static thermomechanical analyzer and subjected to thermal expansion coefficient measurements within a temperature range of 30–700 °C at a heating rate of 5 °C/min under high-purity argon protection. The obtained data are used to calculate the average linear expansion coefficient according to the following Formula (1):

1 $$\alpha = \frac{dL}{{dT}} \cdot \frac{1}{{L_{0} }}$$

where α represents the average linear thermal expansion coefficient, dL represents linear expansion, dT represents the temperature difference, and L_0_ represents the initial length.

A laser cutting machine is used to cut the cladding layer into 30-mm samples, and synchronous oil cooling during the cutting process is used to mitigate the impact of heat on the structure. The surface of the sample coating is cleaned, and surface cracks in the 30-mm cladding layer are observed under optical microscopy (Zeiss Vert.). Another group of cladding samples with dimensions of 20 × 20 × 10 mm is cut using the same method. The cross section of the coating is cleaned and polished before being corroded with 20% nitric acid alcohol for 20 s. The microstructure of the cladding layer is observed using a Zeiss Vert. A1 metallographic microscope and a Tesan VEGAII lMH scanning electron microscope. Energy spectrum analysis of the coating structure is conducted using a Penta EFT- × 3 spectrometer. A D8-ADANCE X-ray diffraction analyzer is used to analyze the phase of the coating and test the residual stress. The microhardness of the cladding layer is measured using an HV-1000IS hardness tester with a load of 200 g and a loading time of 10 s. Dry sliding wear tests of the cladding layer are conducted for 30 min using an M-2000 friction and wear tester with a load of 300 N and a spindle speed of 240 r/min. Prior to the wear test, the cladding layer is preground for 5 min, and ultrasonic cleaning is performed to remove wear debris and enhance the test accuracy. A Tesan VEGAII lMH scanning electron microscope is used to determine the morphology of the worn surface of the cladding layer.

## Results and discussion

### Analysis of the coefficient of expansion of cladding layer

The linear thermal expansion coefficient characterizes the increase in length per degree Celsius of a material due to temperature changes, serving as a significant indicator of the Joule effect^18^. The main factors affecting the linear thermal expansion coefficient of a coating are the composition of alloying elements and the phase transitions that occur during the experimental process. By adding varying contents of Al to Invar alloys, their performance can be enhanced while preserving their low coefficients of thermal expansion. Table 3 shows the coefficients of thermal expansion of the composite coatings at different temperature ranges with varying Al concentrations. Table 3 shows that the thermal expansion coefficient of the Invar alloy coating without Al is the lowest across the different temperature ranges. Coating layers with 2% Al and 4% Al exhibit increases in their thermal expansion coefficients across different temperature ranges, yet they remain lower than those of the 45 steel base. Coating layers with 6% Al and 8% Al exhibit rapid increases in their thermal expansion coefficients across different temperature ranges, surpassing that of the 45 steel base. This difference likely arises due to the relatively high thermal expansion coefficient of in situ precipitated metallic compounds, which significantly affects the thermal expansion coefficient of the composite coating.

**Table 3:** Average coefficients of thermal expansion for different temperature ranges α (10^–6^/℃) and residual stresses in the bonding zone of the fusion layer.

Sample (wt%)	Average coefficient of expansion across different temperature ranges (10^–6^/°c)				Residual stress (MPa)
	30–100 (°C)	30–300 (°C)	30–500 (°C)	30–700 (°C)	Bond zone
45 steel	11.19	13.04	14.33	14.88	–
Invar alloy	1.45	5.88	9.78	11.23	201
2% Al	4.55	9.73	11.78	13.11	173
4% Al	5.11	10.21	13.78	14.61	138
6% Al	7.43	13.66	15.34	18.11	218
8% Al	8.45	14.22	17.33	19.43	242

Due to the differences in the coefficients of thermal expansion, overlay materials and substrates expand at different rates during heating. Overlay materials may expand rapidly, while the substrate expands according to its own thermal expansion coefficient. This nonuniform expansion can result in residual compressive stresses within the overlay^19^. The residual stress at the junction of the laser cladding coating and substrate is measured. Table 3 shows that when the Al content is 4%, the residual compressive stress reaches the minimum value of 138 MPa. The presence of residual compressive stress may alter the grain size and orientation of the cladding layer, thus creating microcracks and defects. These changes not only affect the mechanical properties of the cladding layer but also reduce the wear resistance and fatigue resistance^20^.

### Macroscopic analysis of coatings

**Figure 3:**
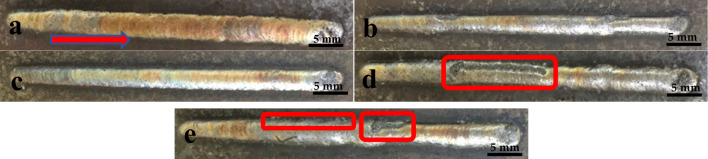
Surface macrostructures of alloys with different Al contents: (**a**) Invar alloy, (**b**) 2% Al, (**c**) 4% Al, (**d**) 6% Al, and (**e**) 8% Al. (When the Al content is 4%, the macroscopic morphology of the fusion coating is optimal).

Figure 3 shows the macroscopic morphologies of the overlay coatings at various Al concentrations. The graph shows that when the Al content is less than 4%, the morphology of the overlay coating is relatively smooth and uniform. The optimal macroscopic morphology of the overlay coating is achieved at an Al content of 4%. However, when the Al content increases to 6%, impurity particles, edge balling, and increased surface roughness are observed in the overlay coating. At an Al content of 8%, the overlay coating exhibits additional impurities, severe balling, weakened metallurgical bonding at the edge with the substrate, and faint macroscopic cracks on the coating surface. Additionally, the width of the overlay coating slightly increases in the direction indicated by the arrows. The measurements show that the width of each coating increases in the range of 0.5–0.8 mm. When the Al content is low, the overlay coating exhibits the Invar effect, with a low overall thermal expansion coefficient similar to that of the base material, resulting in relatively few defects in the overlay coating. As the Al content increases, the thermal expansion coefficient of the overlay material increases, leading to great differences between the coating and the substrate. This phenomenon results in intense metal compound reactions under laser irradiation, preventing the formation of a smooth and continuous surface coating on the overlay coating^21^. The variation in coating width occurs due to the gradual absorption and accumulation of heat by the substrate and the already overlayed layers during the continuous laser cladding process. This thermal accumulation effect leads to a gradual increase in the temperature of the molten pool, thereby enhancing fluidity and slightly increasing coating width during the latter half of the laser cladding process.

The laser cladding dilution rate represents the extent of diffusion of the base material in the coating, which significantly influences its functionality. An excessively high dilution rate can lead to significant losses in the base material and result in the excessive dilution of elements from the base material into the covering layer, severely affecting the hardness and friction of the coating. The formula is as follows:

2 $$\eta = \left( {h/h + H} \right) * 100\%$$

where η is the dilution rate of the overlay coating, H is the height of the overlay coating (μm), and h is the depth of the molten pool (μm).

**Figure 4:**
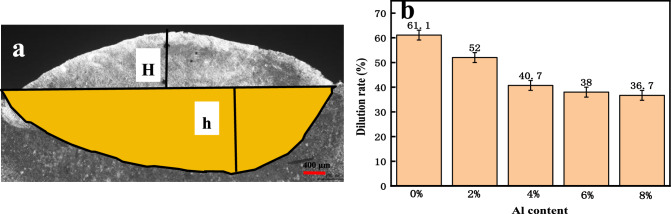
Dilution rates of coatings with different Al contents: (**a**) schematic diagram for calculating the dilution rate of the Invar alloy (0% Al) and (**b**) graph illustrating the trend in the dilution rate. (As the Al content increases, the dilution of the cladding layer first rapidly decreases and then stabilizes).

The influence of varying the Al content on the dilution rate of the overlay specimens is illustrated in Fig. 4. With increasing Al content, the dilution rate of the overlay coating decreases rapidly before stabilizing. At an Al content of 4%, the dilution rate of the overlay specimen is 40.07%. This result is attributed to the increasing energy required for sintering coatings of the same thickness with the gradual addition of Al, decreasing the temperature in the molten pool and thus reducing the dilution rate.

### Analysis of the cladding layer cracks

**Figure 5:**
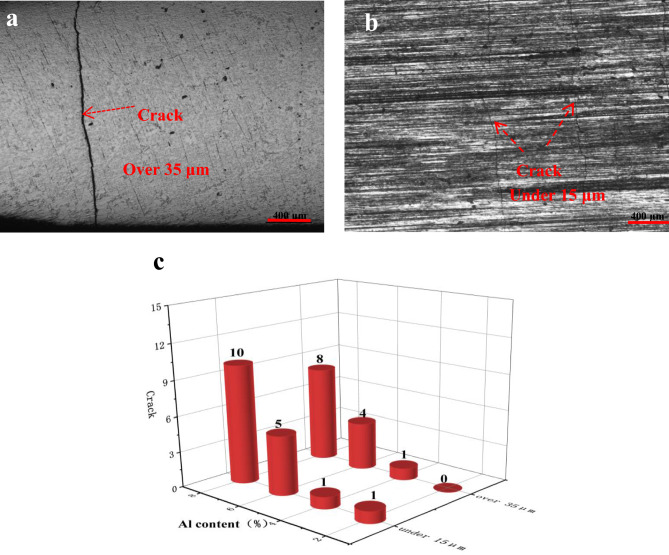
Surface cracks in the cladding layer: (**a**) over 60 μm crack (6%Al), (**b**) under 15 μm crack (6%Al), and (**c**) surface crack under different Al contents. (As the Al content increases, the number of cracks in the coating gradually increases. At an Al content of 4%, there is one crack with a width exceeding 60 μm and one crack with a width below 15 μm).

The macroscopic crack morphology is observed through OM, as shown in Fig. 5. The cladding cracks are oriented perpendicular to the direction of the laser scanning speed, appearing as stripes that traverse the entire cladding layer. For cladding layers with different Al contents, the number of cracks with widths greater than 35 μm and less than 15 μm is counted across the same length (30 mm). To ensure the accuracy of the experiment, 5 sets of crack statistics are recorded for fusion coatings with the same Al content, and the average value is measured at the end. With an increase in the Al content, the number of cracks gradually increases. At an Al content of 2%, the cladding layer has only one crack below 15 μm. At an Al content of 4%, one crack with a width above 35 μm and one with a width below 15 μm appears. As the Al content increases to 6%, the total number of cracks in the coating reaches 9. At an Al content of 8%, the total number of cladding layer cracks greatly increases to 18. These results are analyzed as follows. When the Al content is low, the cladding layer exhibits the Invar effect, with a low coefficient of thermal expansion resulting in minimal stress during cladding, thereby reducing the formation of cracks. However, when the Al content exceeds a certain threshold, the excess Al disrupts the Invar effect. The expansion coefficient of the cladding material increases, resulting in an increase in tensile stress during cladding, and stress release generates cracks.

### Microstructure of the cladding layer

**Figure 6:**
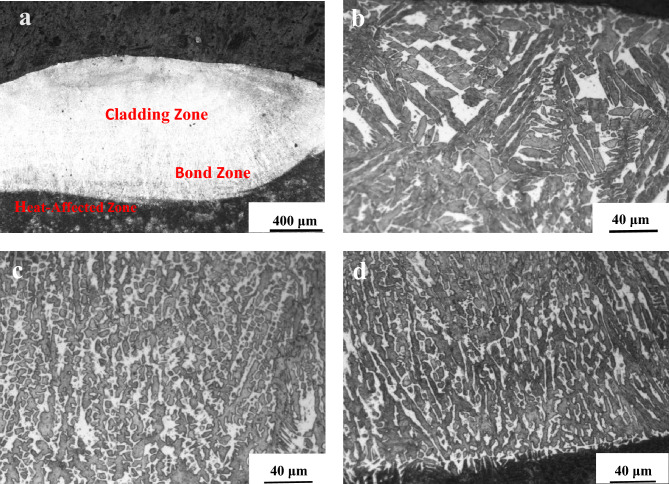
Microstructure of the Fe–Ni–Al coating with 4% Al: (**a**) macrostructure, (**b**) upper portion of the coating, (**c**) middle portion of the coating, and (**d**) bottom portion of the coating. (The upper part of the overlay coating consists of columnar crystals with grain sizes of approximately 50 μm; the middle part contains equiaxed crystals with grain sizes of approximately 6 μm; and the bottom part contains elongated dendritic crystals with sizes ranging from 10 to 20 μm).

Figure 6 shows a micrograph of the metallographic structure of the overlay coating with a 4% Al content. This figure illustrates the transition of the structure from the surface to the substrate, including the overlay zone (CL), the bond zone (BZ), and the heat-affected zone (HAZ)^22^. There is a distinct interface between the overlay coating and the heat-affected zone, indicating metallurgical bonding between the substrate and the overlay coating. Figure 6 shows that the upper part of the overlay coating consists of columnar crystals with grain sizes of approximately 50 μm. The middle part exhibits equiaxed crystals, a dense structure and grain sizes of approximately 6 μm. Finally, the bottom part displays elongated dendritic crystals with sizes ranging from 10 to 20 μm. The crystalline morphology of the overlay coating is influenced by the temperature gradient and solidification rate. At the bottom of the overlay coating, due to large temperature gradients, grains grow epitaxially attached to the substrate, resulting in coarse columnar crystals. In the middle of the overlay coating, the grains form fine equiaxed crystals under rapid cooling and heating conditions. At the top of the overlay coating, increased solidification rates due to direct contact with air lead to the rapid growth of the structure into columnar and dendritic shapes^23^.

**Figure 7:**
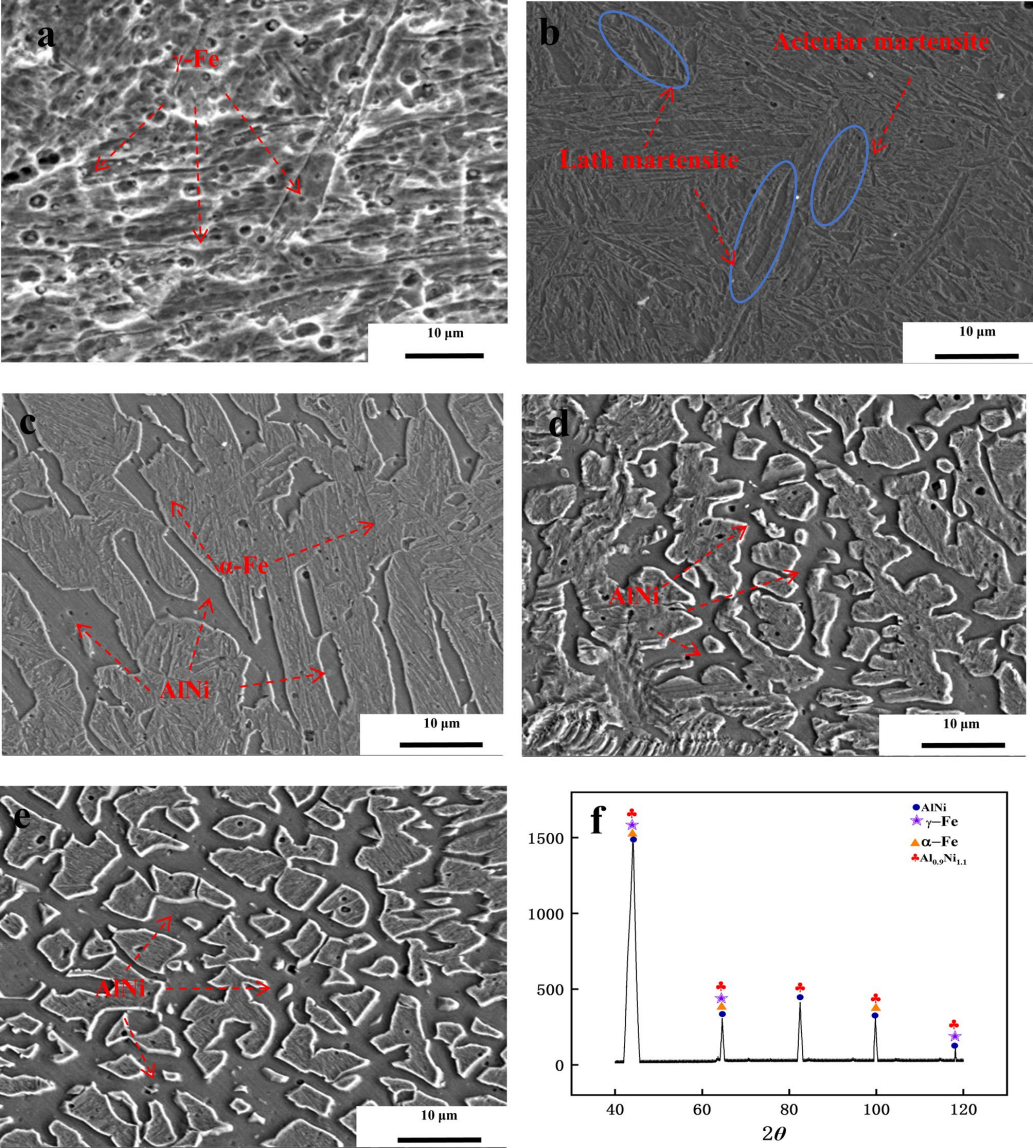
Microstructural characterization of the deposited layers with varying Al contents: (**a**) Invar alloy; (**b**) 2% Al; (**c**) 4% Al; (**d**) 6% Al; (**e**) 8% Al; and (**f**) XRD diffraction pattern of the Fe–Ni–Al coating with 4% Al. (As the Al content increases, the amount of AlNi intermetallic compounds gradually increases. These intermetallic compounds intertwine with each other, forming a network-like structure. The overlay coating mainly contains AlNi and Al_0.9_Ni_1.1_ intermetallic compounds and martensite and ferrite phases generated from the γ‒Fe phase transition to α–Fe).

**Figure 8:**
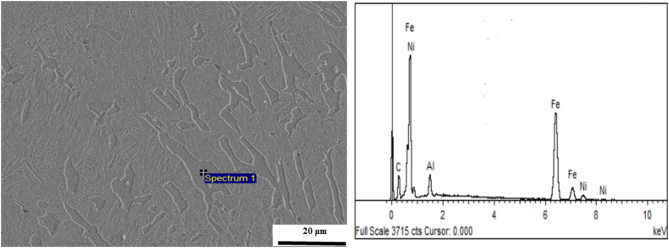
Energy spectrum analysis of the Fe–Ni–Al overlay coating with 4% Al. (The energy spectrum of the Fe–Ni–Al overlay coating indicates the formation of AlNi intermetallic compounds.)

Figure 7 shows the microstructures of the overlay coatings with varying Al contents under scanning electron microscopy (SEM). Figure 7a shows the microstructure of the Invar alloy, where the overlay structure consists of [Fe, Ni] austenite formed by nickel dissolved in γ-Fe. The solid solution phase exhibits uniform distribution characteristics without apparent precipitates or second-phase aggregation. Figure 7b shows the overlay with 2% Al. The introduction of Al leads to the phase transformation of the original austenite structure into lamellar and needle-like martensite. Aluminum exists in the matrix in the form of a small amount of solid solution. With an Al content of 4%, a small proportion of intermetallic compounds is formed. According to the energy spectrum in Fig. 8, the atomic ratio of Al to Ni is 1:1, indicating that the intermetallic compound formed in the overlay coating is AlNi. These intermetallic compounds are dispersed in the matrix phase. Additionally, at this point, a martensitic phase transformation occurs in the structure, but small amounts of residual austenite are still present. With an Al content of 6%, the number and size of intermetallic compounds may significantly increase. These phases form complex cluster structures, which significantly impact the mechanical and corrosion resistance properties of the overlay coating. At this point, the overlay coating loses its Invar effect. With 8% Al, many intermetallic compounds are present in the overlay coating, forming an intertwined network structure^24^.

Figure 7f shows the XRD pattern of the overlay coating with 4% Al. The XRD pattern shows that the overlay coating mainly contains AlNi and Al_0.9_Ni_1.1_ intermetallic compounds, [Fe, Ni] austenite formed by Ni dissolved in γ-Fe, and martensite and ferrite phases generated from the γ-Fe phase transition to α-Fe. The typical Invar alloy coating exhibits a [Fe, Ni] austenite structure formed by Ni dissolved in γ-Fe. With the addition of Al, the Curie temperature of the material decreases. According to previous research, for every 0.1% increase in the Al content, the Curie temperature of the material decreases by 3 °C^25,26^. As the Al content increases, the Curie temperature of the overlay coating gradually decreases, disrupting the original system of the Fe-36Ni Invar alloy and promoting the transition from the γ phase to the α phase, inducing a martensitic phase transformation.

### Influence of different Al contents on the microhardness of the cladding coating

**Figure 9:**
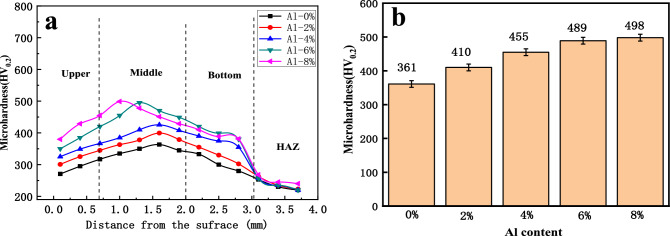
Microhardnesses under different Al contents of the composite coatings: (**a**) microhardness of the different zones and (**b**) average microhardness. (The microhardness of the middle part of the cladding layer is the highest, while the upper and lower parts exhibit relatively low microhardnesses. With increasing Al content, the microhardness of the cladding layer initially increases rapidly before stabilizing).

**Figure 10:**
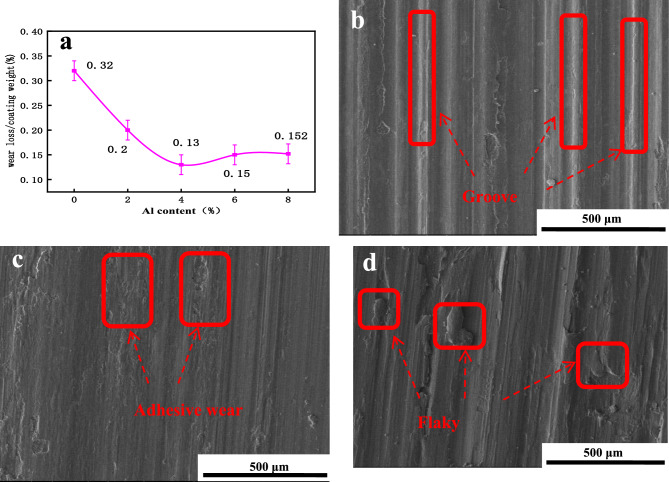
Wear performance of the Fe–Ni–Al cladding layers: (**a**) wear resistance of the layers deposited with different Al contents; (**b**) wear morphology of the Invar alloy coatings; (c) wear morphology of the 4% Al coating; and (d) wear morphology of the 8% Al coating. (When the Al content is 4%, the cladding layer exhibits the best wear resistance. The primary wear mechanism of the cladding layer is adhesive wear, accompanied by abrasive wear).

Figure 9 shows the microhardness of the overlay coatings with varying Al contents. The microhardness varies noticeably among the upper, middle, and lower sections of the overlay coating, with the highest microhardness observed in the middle section. The upper and lower sections exhibit relatively low microhardnesses, with an average microhardness of 250 HV in the heat-affected zone (HAZ). Figure 10b shows the average microhardness of the middle section of the overlay coatings with different Al contents. When the coating lacks Al, as in the case of the Invar alloy overlay coating, the average hardness of the overlay coating is 361 HV. With increasing Al content, the microhardness of the overlay coatings continues to increase. When the Al content reaches 6%, the microhardness of the coating is 489 HV. At this point, the microhardness is 1.36 times greater than that of the Invar alloy coating. Further increasing the Al content does not significantly alter the microhardness of the overlay coating. The upper and lower sections of the overlay coating predominantly exhibit columnar or dendritic structures with coarse grains. The middle section of the overlay coating features fine structures, mainly consisting of equiaxed grains, resulting in a higher microhardness than that in other regions of the overlay coating^27^. The addition of Al significantly increases the microhardness of the overlay coating. This phenomenon is primarily attributed to the change in the original single austenite structure of the overlay coating by Al, which induces a phase transformation where the hardness of martensite increases. Additionally, the reinforcement effect of in situ-formed AlNi intermetallic compounds leads to a rapid increase in the microhardness.

### Influences of different Al contents on the wearability of cladding coatings

The wear resistance of the overlay coating is characterized by the ratio of weight loss due to wear to the total mass. Figure 10a shows the influence of varying the Al content on the wear resistance of the overlay coating. The graph shows that with increasing Al content, the wear resistance of the overlay coating sharply increases before gradually decreasing. When the Al content is 4%, the wear resistance of the overlay coating is optimal and is 2.46 times greater than that of the Invar alloy (0% Al). The wear resistance of an Invar alloy overlay coating is typically moderate. However, upon the addition of Al, the in situ formation of AlNi intermetallic compounds with Ni in the overlay coating results in increased hardness and wear resistance. Hence, the wear resistance of the overlay coating is enhanced. When the addition of Al exceeds a certain range, the interfacial effect of the overlay coating is completely disrupted, leading to unfused particles, cracks, pores, and compositional segregation in the overlay coating. Unfused particles in the coating become stress concentration points when subjected to wear, leading to premature failure of the coating. Pores and cracks in the coating serve as potential fracture sources and are prone to expansion under external forces, resulting in coating delamination and significantly reducing the fatigue life and wear resistance of the coating. Compositional segregation within the coating leads to the formation of regions with varying hardnesses, resulting in stress concentration during wear. Consequently, the coating exhibits phenomena such as granular and chunky spalling during the wear process, leading to a decrease in wear resistance^28^.

Figures 10b–d show the wear morphologies of the coatings with varying Al contents. The figure shows that the overlay coating exhibits adhesive and abrasive wear during the wear process. On the wear surface of the Invar alloy overlay coating, deep furrows are observed, and severe metal cutting occurs on the coating surface. After the addition of Al, adhesive wear becomes the primary wear mechanism of the overlay coating. Consequently, the wear scars on the overlay coating become relatively shallow, and the wear surface of the specimen appears flat. No spalling debris is observed during the wear process, indicating a relatively stable wear process. When there is an excessive amount of Al, the wear morphology of the overlay coating changes. Small flake-like spalling occurs on the surface of the coating, and the wear is highly nonuniform^29^.

Local heating generated during the wear process results in the formation of deep furrows on the overlay coating surface as soft metal is plowed during the sliding process^30^. During the wear of the Invar alloy overlay coating, the relatively low hardness of the overlay coating results in significant plowing on the coating surface during wear, leading to the formation of deep furrows^31^. With the addition of Al, the hardness of the overlay coating increases, resulting in a stable wear surface. When there is an excessive amount of Al, the interfacial effect of the coating is disrupted, resulting in numerous surface cracks within the composite coating. This effect compromises the overall integrity of the composite coating, leading to a decrease in wear resistance.

## Conclusions

In this study, the laser cladding of Fe–Ni–Al composite coatings on 45 steel was investigated, and the low coefficient of thermal expansion resulting from the Invar effect was exploited. The influence of the Al content on the microstructure and properties of the Fe–Ni–Al composite coatings was examined. The following conclusions were drawn:

1. When laser cladding the Fe–Ni–Al composite coatings, the coefficient of thermal expansion of the cladding layer gradually increased with increasing Al content. The number of cracks in the coating increased gradually, while the dilution rate of the coating decreased gradually. When the Al content reached 4%, the coefficient of thermal expansion of the cladding layer was closest to that of the substrate. The residual stress at the junction of the laser cladding coating and substrate reached a minimum value of 138 MPa. At this point, there was one crack wider than 60 μm and one crack narrower than 15 μm in the cladding layer, and the dilution rate of the cladding layer was 40.07%.
2. The upper part of the Fe–Ni–Al cladding layer consisted of columnar grains with grain sizes of approximately 50 μm. The middle part comprised equiaxed grains with grain sizes of approximately 6 μm. Finally, the bottom part consisted of elongated dendrites with dendrite sizes ranging from 10 to 20 μm. When the Al content was 4%, the Fe–Ni–Al composite coating mainly consisted of AlNi and γ-[Fe,Ni] austenite. With increasing Al content, the γ phase transformed into the α phase, and the amount of AlNi intermetallic compounds gradually increased. These intermetallic compounds intertwined to form a network structure.
3. When the Al content was 4%, the average microhardness of the middle part of the Fe–Ni–Al cladding layer was 455 HV. At this point, the wear resistance of the cladding layer was the best, with a wear loss ratio of 0.13% and a wear resistance 2.46 times greater than that of the Invar alloy (0% Al) coating. The main wear mechanism of the coating was adhesive wear accompanied by abrasive wear.

## Data Availability

The datasets generated and analysed during the current study are not publicly available due Laboratory policies or confidentiality agreements but are available from the corresponding author on reasonable request.

## References

[1] Zhu L (2021). Recent research and development status of laser cladding: A review. Opt. Laser Technol..

[2] Zhao L, Zhao M-J, Li D-Y, Zhang J, Xiong G-Y (2012). Study on Fe–Al–Si in situ composite coating fabricated by laser cladding. Appl. Surf. Sci..

[3] Fu F, Zhang Y, Chang G, Dai J (2016). Analysis on the physical mechanism of laser cladding crack and its influence factors. Optik.

[4] Zhou S, Zeng X, Hu Q, Huang Y (2008). Analysis of crack behavior for Ni-based WC composite coatings by laser cladding and crack-free realization. Appl. Surf. Sci..

[5] Shiga M (1996). Invar alloys. Curr. Opin. Solid State Mater. Sci..

[6] Zhan X, Qi C, Gao Z, Tian D, Wang Z (2019). The influence of heat input on microstructure and porosity during laser cladding of Invar alloy. Opt. Laser Technol..

[7] Schlosser WF (1971). A model for the invar alloys and the Fe−Ni system. J. Phys. Chem. Solids.

[8] Zhu S (2024). Analysis of the thermal expansion and mechanical properties of laser cladding of Invar alloy. Int. J. Adv. Manuf. Technol..

[9] Sahoo A, Medicherla VRR (2021). Fe-Ni invar alloys: A review. Mater. Today Proc..

[10] Ha TK, Lee KD, Song JH, Jeong HT (2007). Effect of aging treatment conditions on the microstructure and strength of Fe-36Ni based invar alloy. KEM.

[11] Sui Q (2019). Strengthening of the Fe-Ni invar alloy through chromium. Materials.

[12] Sakaguchi N, Ohno H, Nakada N (2022). Strengthening of super invar cast steel by precipitation of intermetallic compounds. ISIJ Int..

[13] Vinogradov A, Hashimoto S, Kopylov VI (2003). Enhanced strength and fatigue life of ultra-fine grain Fe–36Ni invar alloy. Mater. Sci. Eng. A.

[14] Kim S-H, Kim H, Kim NJ (2015). Brittle intermetallic compound makes ultrastrong low-density steel with large ductility. Nature.

[15] Shi D, Wen B, Melnik R, Yao S, Li T (2009). First-principles studies of Al–Ni intermetallic compounds. J. Solid State Chem..

[16] Zhang Y, Jin K, Li Z, Wei S, Guo J (2023). Microstructures, wear and corrosion behaviors of laser cladding in situ synthetic Al_3_Ti/AlNi/AlNi_3_/MgNi_2_ composite coatings on magnesium alloy using Al as middle layer. J. Mater. Eng. Perform..

[17] Perrot-Simonetta MT, Kobylanski A (1995). Influence of trace elements on hot ductility of an ultra high purity invar alloy. J. Phys. IV Fr..

[18] Watanabe H, Yamada N, Okaji M (2004). Linear thermal expansion coefficient of silicon from 293 to 1000 K. Int. J. Thermophys..

[19] Arsenault RJ, Taya M (1987). Thermal residual stress in metal matrix composite. Acta Metall..

[20] Wang YD, Lin Peng R, Wang X-L, McGreevy RL (2002). Grain-orientation-dependent residual stress and the effect of annealing in cold-rolled stainless steel. Acta Mater..

[21] Manoj A (2023). Surface modification of grey cast iron by laser cladding for automotive brake disc application. Wear.

[22] Gong N (2023). Laser-cladding of high entropy alloy coatings: An overview. Mater. Techno..

[23] Wegener T (2021). On the structural integrity of Fe-36Ni invar alloy processed by selective laser melting. Addit. Manuf..

[24] Ma K (2014). Mechanical behavior and strengthening mechanisms in ultrafine grain precipitation-strengthened aluminum alloy. Acta Mater..

[25] Chen D, Harward I, Baptist J, Goldman S, Celinski Z (2015). Curie temperature and magnetic properties of aluminum doped barium ferrite particles prepared by ball mill method. J. Magnet. Magnet. Mater..

[26] Nelson J, Sanvito S (2019). Predicting the Curie temperature of ferromagnets using machine learning. Phys. Rev. Mater..

[27] Devojno OG, Feldshtein E, Kardapolava MA, Lutsko NI (2019). On the formation features, structure, microhardness and tribological behavior of single tracks and coating layers formed by laser cladding of Al-Fe powder bronze. Surf. Coat. Technol..

[28] Zhang Z, Chen D (2006). Consideration of Orowan strengthening effect in particulate-reinforced metal matrix nanocomposites: A model for predicting their yield strength. Script. Mater..

[29] Molinari J-F, Aghababaei R, Brink T, Frérot L, Milanese E (2018). Adhesive wear mechanisms uncovered by atomistic simulations. Friction.

[30] Mishina H, Hase A (2013). Wear equation for adhesive wear established through elementary process of wear. Wear.

[31] Chen YC, Liao YS (2003). Study on wear mechanisms in drilling of Inconel 718 superalloy. J. Mater. Process. Technol..

